# Virus infection induced pulmonary fibrosis

**DOI:** 10.1186/s12967-021-03159-9

**Published:** 2021-12-07

**Authors:** Wei Jie Huang, Xiao Xiao Tang

**Affiliations:** 1 State Key Laboratory of Respiratory Disease, National Clinical Research Center for Respiratory Disease, National Center for Respiratory Medicine, Guangzhou Institute of Respiratory Health, The First Affiliated Hospital of Guangzhou Medical University, Guangzhou, China; 2 Guangzhou Laboratory, Bio-island, Guangzhou, China

**Keywords:** Virus infection, Pulmonary fibrosis, Mechanisms, SARS-CoV-2, Potential anti-fibrotic therapy

## Abstract

Pulmonary fibrosis is the end stage of a broad range of heterogeneous interstitial lung diseases and more than 200 factors contribute to it. In recent years, the relationship between virus infection and pulmonary fibrosis is getting more and more attention, especially after the outbreak of SARS-CoV-2 in 2019, however, the mechanisms underlying the virus-induced pulmonary fibrosis are not fully understood. Here, we review the relationship between pulmonary fibrosis and several viruses such as Human T-cell leukemia virus (HTLV), Human immunodeficiency virus (HIV), Cytomegalovirus (CMV), Epstein–Barr virus (EBV), Murine γ-herpesvirus 68 (MHV-68), Influenza virus, Avian influenza virus, Middle East Respiratory Syndrome (MERS)-CoV, Severe acute respiratory syndrome (SARS)-CoV and SARS-CoV-2 as well as the mechanisms underlying the virus infection induced pulmonary fibrosis. This may shed new light on the potential targets for anti-fibrotic therapy to treat pulmonary fibrosis induced by viruses including SARS-CoV-2.

## Introduction

**Fig. 1:**
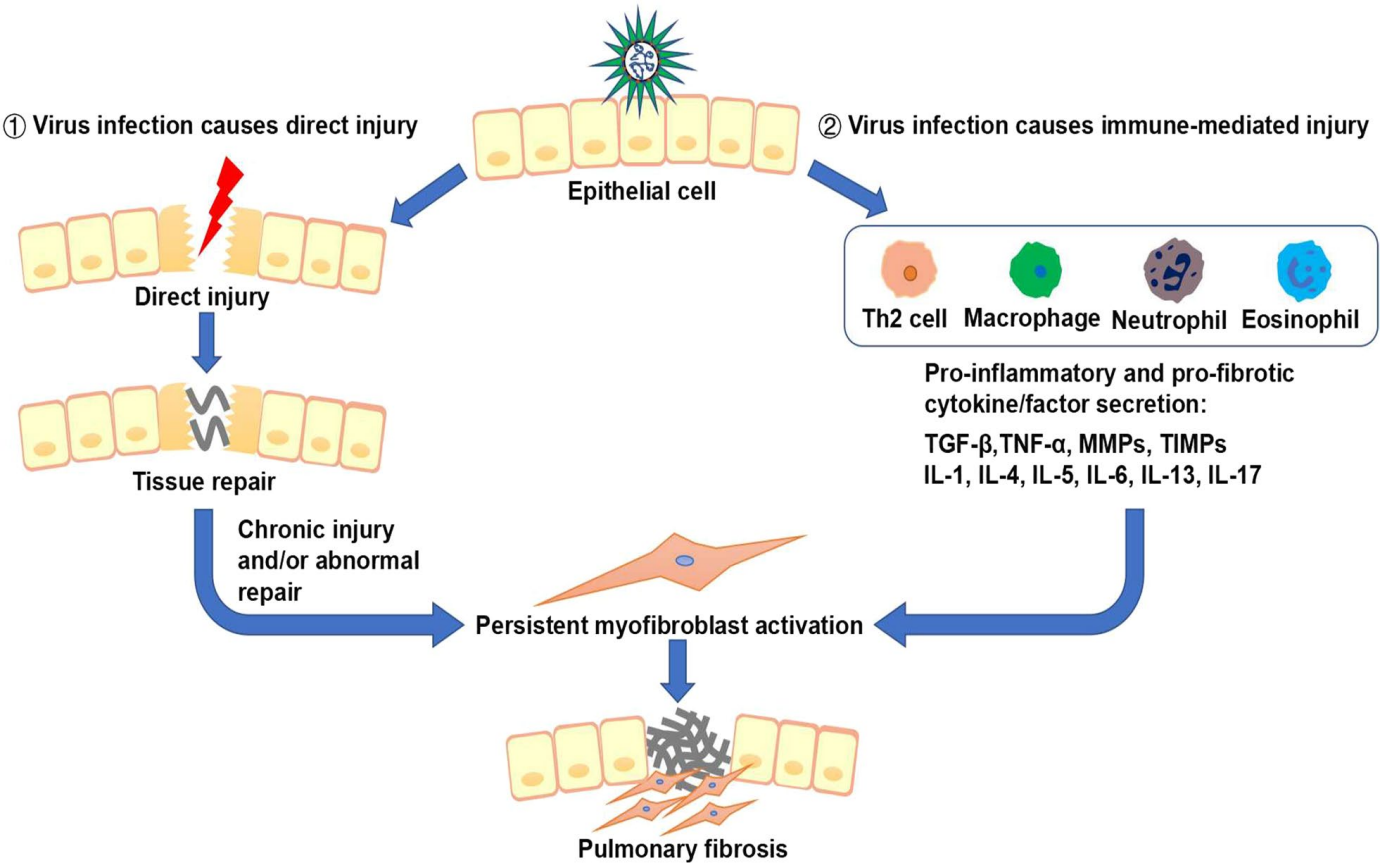
Two main pathways of virus-induced injury and pulmonary fibrosis. ① Viral infection causes direct damage to the lung. During most viral infections, the virus causes prompt and direct damage to the lung. Wound healing response is activated at this time, however, the virus induces persistent lung damage and/or abnormal wound-healing, leading to occurrence of pulmonary fibrosis. ② Viral infection causes immune-mediated injury. Virus infection activates the immune system. Macrophages, neutrophils, eosinophils, and Th2 cells aggregate at the site of injury and release a large number of pro-inflammatory and pro-fibrotic cytokines/factors such as TGF-β, TNF-α, MMPs, TIMPs, IL-1, IL-4, IL-5, IL-6, IL-13 and IL-17. The combination of the virus and these factors induces sustained and substantial lung damage, promoting pulmonary fibrosis

Pulmonary fibrosis occurs as a consequence of many types of severe lung injury and is largely associated with inflammatory responses. Alveolar inflammation is important for amplifying host defenses in the lung, and alveolar macrophages contribute to this response [1]. Viruses may induce pulmonary fibrosis through the following two pathways. ① Viral infection causes direct damage to the lung. During most viral infections, the virus causes prompt and direct damage to the lung. Wound healing response is activated at this time, however, the virus induces persistent lung damage and/or abnormal wound-healing, leading to development of pulmonary fibrosis. ② Viral infection causes immune-mediated injury. After injury, the lung is in a state of inflammatory infiltration and the virus infection activates the immune system. Macrophages, neutrophils, eosinophils, and Th2 cells aggregate at the site of injury and release a large number of pro-inflammatory and pro-fibrotic cytokines/factors such as transforming growth factor-β (TGF-β), tumor necrosis factor-α (TNF-α), matrix metalloproteinases (MMPs), tissue inhibitor of metalloproteinases (TIMPs), Interleukin (IL)-1, IL-4, IL-5, IL-6, IL-13 and IL-17. The combination of the virus and these factors induces sustained and substantial lung damage, promoting pulmonary fibrosis (Fig. 1). The prognosis of patients with pulmonary fibrosis induced by viral infection varies. Some die eventually, while some get favorable prognosis. More than one-third of patients who survived severe COVID-19 pneumonia discharged from the hospital developed pulmonary fibrosis [2]. Moreover, many studies have reported that viruses such as CMV, Influenza virus, Avian influenza virus, SARS-CoV, MERS-CoV cause long-term damage to the lung and remain as risk factors for pulmonary fibrosis for a long time after infection. Here we review the relationship between a number of viral infections and pulmonary fibrosis, and also summarize the underlying mechanisms as well as future research directions which may shed new light on the therapeutic strategies against pulmonary fibrosis induced by viruses including SARS-CoV-2.

### Human T-cell leukemia virus

HTLV type-I, the first reported human oncogenic retrovirus, is the etiologic agent of adult T-cell leukemia lymphoma (ATL) and HTLV-I-associated myelopathy (HAM)/tropical spastic paraplegia (TSP) [3]. The lung is one of the organs affected by HTLV-1-mediated inflammation, which involves interstitial pneumonias, bronchiolitis and alveolitis. In a computed tomography (CT) study of 106 HTLV positive patients, 65 (61.3%) patients had abnormal CT findings, including ground glass opacities (31.1%), bronchiectasis (26.4%), centrilobular nodules (23.6%), septal thickening (17.9%), honeycombing (4.7%) and crazy-paving appearance (2.8%) [4]. Two CT studies suggested that pulmonary fibrosis occurs in patients with HTLV [4, 5] (Table 1). The viral transactivator protein Tax of HTLV-I activates many cellular genes such as TGF-β, which may be an important factor of HTLV-I-induced pulmonary fibrosis [6]. Teruya et al. showed that HTLV-I infected lung epithelial cells and stimulated them to produce cytokines, chemokines, and cell adhesion molecules through induction of NF-κB and activator protein (AP)-1, contributing to the clinical features of HTLV-I-related pulmonary diseases [7]. Matsuyama et al. demonstrated that HTLV-I positive patients with cryptogenic fibrosing alveolitis (CFA) significantly increased MMP-2 level in bronchoalveolar lavage fluid (BALF) as compared to HTLV-I negative patients, and no differences in the TIMP-2, indicating that the imbalance of MMPs and TIMPs maybe the cause of HTLV-induced pulmonary fibrosis. They also noticed increased expression of macrophage inflammatory protein (MIP)-1α, interferon-γ inducible protein (IP)-10 and soluble intercellular adhesion molecule (sICAM) in BALF [8]. Miyazato et al. showed that HTLV-I infection leads to clear expression of inflammatory cytokines such as IL-1β, TNF-α and interferon (IFN)-γ as well as chemokines including regulated upon activation normal T-cell expressed and secreted (RANTES), monocyte chemoattractant protein 1 (MCP-1), MIP-1α and IP-10 in the lung of transgenic mice at mRNA level [9] (Table 2).

**Table 1:** Clinical manifestation summary of pulmonary fibrosis induced by viruses

Virus	Clinical manifestation	Assessment of fibrosis	Reversibility of pulmonary fibrosis	References
Human T-cell leukemia virus	ATL, leukemic cell infiltration, pulmonary fibrosis	CT showed ground glass opacities, bronchiectasis, centrilobular nodules, septal thickening, honeycombing and crazy-paving, suggesting the presence of pulmonary fibrosis	No mention	Assessment:[4]; reversibility: None
Human immunodeficiency virus	Interstitial pneumonia, pulmonary fibrosis	HRCT demonstrated areas of ground glass opacification, consolidation and honeycombing, with interstitial infiltrate as the histopathologic feature	No mention	Assessment:[10]; reversibility: None
Cytomegalovirus	Interstitial pneumonia, pulmonary fibrosis	HRCT demonstrated bilateral mixed areas of ground-glass opacity, poorly-defined centrilobular small nodules, and consolidation	No mention	Assessment:[22]; reversibility: None
Epstein–Barr virus	Unspecific interstitial lung disease, pulmonary fibrosis	The open-lung biopsy showed uncharacteristic focal interstitial peribronchial infiltration in the left lower lobe, with histiocytes and lymphocytes as well as interstitial fibrosis and increased collagen tissue	Reversible: After 26 months, chest X-ray showed only slight interstitial markings	Assessment:[29]; reversibility: [29]
Influenza virus	ARDS, DAD bronchoalveolar pneumonia, pulmonary fibrosis	Histologic features included bronchoalveolar pneumonia, interstitial septal thickening, type II pneumonocyte hyperplasia, fibrosis and squamous metaplasia. HRCT demonstrated ground glass opacity and consolidation	Reversible: The one month follow-up CT scans showed that the fibrosis resolved	Assessment:[36, 37]; reversibility: [37]
Avian influenza virus	ARDS, lymphopenia, pulmonary fibrosis	CT findings in H5N1 and H7N9 patients were ground-glass opacities and lobar consolidation	Reversible: The 12th month follow-up CT of patient showed only minimal residual fibrous lines	Assessment:[44, 46]; reversibility: [44]
MERS-CoV	ARDS, multi-lobar airspace disease, pulmonary fibrosis	CT showed multi-lobar airspace disease, ground-glass opacities and pleural effusions	No mention	Assessment:[57]; reversibility: none
SARS-CoV	ARDS, DAD, pulmonary fibrosis	The histopathological findings were extensive edema, hyaline membrane formation, alveolar collapse, and alveolar epithelial desquamation. CT showed ground-glass opacities and consolidation	Reversible: HRCT scan showed improvement of pulmonary fibrosis in one month	Assessment:[67, 68, 71]; reversibility: [69, 72]
SARS-CoV-2	ARDS, DAD, pulmonary fibrosis	Histopathological examination of the lung biopsy tissues revealed bilateral acute changes with DAD, reactive type II pneumocyte and macrophage hyperplasia, patchy inflammatory cellular infiltration and loose interstitial fibrosis	Reversible: Thin-section chest CT showed that pulmonary fibrosis developed in COVID-19 patients could reverse in about a third of the patients 120 days after the onset	Assessment:[88]; reversibility: [86]

**Table 2:** Summary of potential mechanisms underlying pulmonary fibrosis induced by viruses

Virus	Animal model	Manifestation	Potential mechanism	References
Human T-cell leukemia virus	Transgenic mice with HTLV-I	HTLV-I infection causes inflammatory changes in the lung	HTLV-I infection leads to clear expression of inflammatory cytokines such as IL-1β, TNF-α and IFN-γ as well as chemokines including RANTES, MCP-1, MIP-1α and IP-10	[9]
Human immunodeficiency virus	HIV-1 TG mice	HIV related protein gp120 augments α-SMA expression and myofibroblast differentiation in mouse primary lung fibroblasts, promoting pulmonary fibrosis	HIV infection increases α-SMA expression and fibroblast-to-myofibroblast transdifferentiation via CXCR4-ERK1/2 signaling pathway	[13]
Cytomegalovirus	Immunocompetent BALB/c mice	MCMV-infected mice had CMV reactivation 2 weeks after CLP. Compared to the control group, the mRNA of TNF-α, IL-1β, KC and MIP-2 significantly increased and pulmonary fibrosis was also developed in the infected mice	CMV infection altered the expression of TNF-α, IL-1β, KC and MIP-2	[18]
	Bleomycin BALB/c mice	MCMV aggravated pulmonary fibrosis in the bleomycin-treated mice, but not in the control mice	CMV infection induces TGF-β secretion from various cells as well as myofibroblast formation	[24]
	CMV-positive patients	Telomere attrition was exacerbated in CMV-positive individuals	CMV reduces telomere length	[27]
Murine γ-herpesvirus 68	IFN-γ receptor-deficient mice	MHV-68 infection causes epithelial damage, inflammatory response, collagen accumulation and gradually evolves into progressive interstitial fibrosis	MHV-68 infection increases TGF-β and IL-13 expression, as well as imbalance of Th1 and Th2 cytokines	[33]
Influenza	Male albino mice	Pulmonary fibrosis occurred after H1N1 infection, and the volume density of fibrous connective tissue in the lung interstitial increased 9–9.4-fold as compared to the control	H1N1infection increases TGF-β expression, activates the Smad system and triggers EMT	[41]
Avian influenza	C57Bl/6 mice	Pulmonary interstitial fibrosis was observed on the first day post H5N1 infection. And the histological studies showed necrotic foci and atelectasis, inflammatory infiltrates, bleeding, vascular thrombosis, interstitial and alveolar edema	H5N1 infection increases the expression of TNF-α, FGF and EGF, fibroblast proliferation, collagen accumulation and ECM deposition	[50, 51]
MERS-COV	hDPP4 transgenic mice	hDPP4-Tg mice exhibited irregular arrangement of pneumocytes, alveolar septal changes, and inflammatory cell infiltration into the lung. In addition, the lung damage became severe with progressive pulmonary fibrosis, including alveolar septal thickening and macrophage infiltration	MERS-COV infection increases the expression of TNF-α, IL-1β, TGF-β, as well as type I and type III collagen	[63]
SARS-CoV	BALB/c mice	SARS-CoV-infected mice presented DAD and hyaline membrane formation	Although pulmonary fibrosis was not examined in this model, SARS-CoV infection significantly increased the pro-fibrotic cytokines by regulating RAS system	[73]
	C57BL/6J	SARS-CoV-infected mice presented pulmonary interstitial thickening, inflammatory infiltration, DAD, and pulmonary fibrosis	SARS-CoV infection increases the expression of IL-1β, TNF-α, IL-6, TGF-β, CTGF, PDGF and PAI-1	[75]
SARS-CoV-2	Rhesus	SARS-CoV-2-infected rhesus presented interstitial pneumonia. The alveolar interstitial space was greatly expanded by edema, fibrin, macrophages and neutrophils	Although pulmonary fibrosis was not examined in this study, SARS-CoV-2 infection increased the expression of collagen, proinflammatory and pro-fibrotic cytokines	[91]
	Rhesus	SARS-CoV-2-infected rhesus presented interstitial pneumonia. Thickened alveolar walls were observed, with infiltrations of a large number of monocytes and lymphocytes, and a few eosinophils. In the severe lesion area, alveolar wall necrosis, collapse, fibrosis and extensive fibroblast proliferation can be seen	No mechanism has been shown in this study	[92]

### Human immunodeficiency virus

**Fig. 2:**
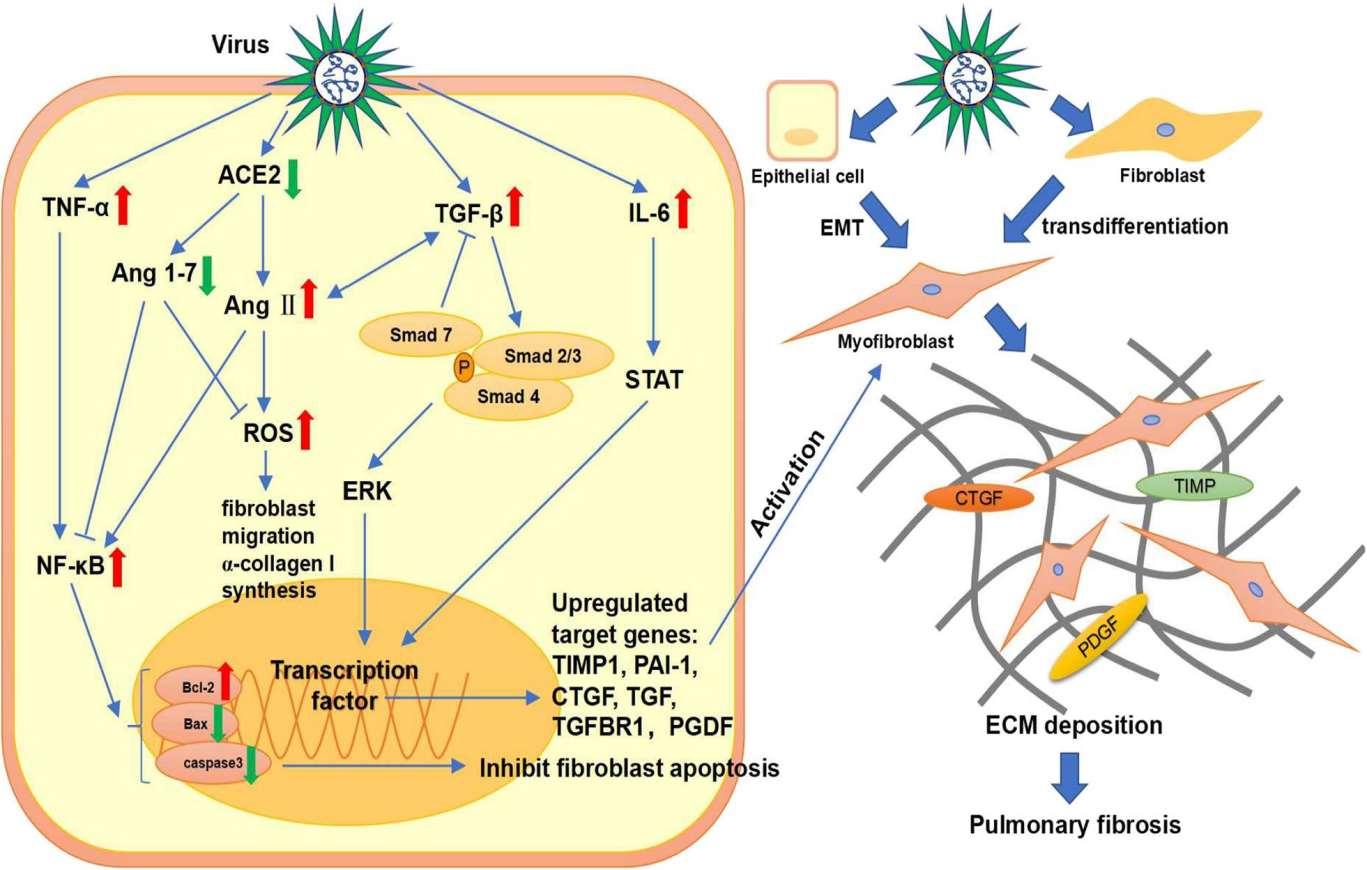
Signaling pathways of pulmonary fibrosis induced by the virus. Virus infection increases the expression of pro-fibrotic and pro-inflammatory cytokines such as TGF-β, TNF-α and IL-6 to promote pulmonary fibrosis. During this process, TGF-β/Smad, ERK and STAT pathways are activated and then upregulate profibrotic cytokines such as TIMP1, PAI-1, CTGF, TGF, TGFBR1, and PGDF. Besides, the decrease in ACE2 upregulates Ang II, leading to increased expression of NF-κB and ROS. NF-κB inhibits fibroblast apoptosis by upregulating Bcl-2 as well as downregulating Bax and caspase 3 to promote pulmonary fibrosis. ROS induces fibroblast migration and α-collagen I synthesis. Moreover, Ang II and TGF-β mutually reinforce each other. Eventually, a large number of myofibroblasts accumulate through EMT and fibroblast transdifferentiation, resulting in extracellular matrix deposition and pulmonary fibrosis

Pulmonary disease, a common complication of HIV infection, often presents as interstitial pneumonia. The high-resolution computerized tomography (HRCT) of HIV-induced interstitial lung diseases demonstrated areas of ground glass opacification, consolidation and honeycombing, with interstitial infiltration as the histopathologic feature [10] (Table 1). Crothers et al. showed that patients infected with HIV had a higher probability of developing pulmonary fibrosis as compared to uninfected people [11]. Additionally, Leader et al. found that 29.4% of the HIV patients had at least a trace level of fibrosis-like change in the lung, and this change had a significant positive correlation with HIV viral load and smoking status [12]. However, it is not known whether HIV-induced pulmonary fibrosis is progressive or stable due to lack of follow-up study. In the basic research, Marts et al. found that HIV protein gp120 induces the expression of α-smooth muscle actin (α-SMA) and transdifferentiation of fibroblasts to myofibroblasts by activating the CXCR4-ERK1/2 signaling pathway in mouse primary lung fibroblasts to promote pulmonary fibrosis. At the same time, they noticed that the lungs of HIV transgenic mice showed an age-dependent increase in hydroxyproline, an amino acid needed for collagen synthesis [13] (Table 2). This may indicate the mechanism of pulmonary fibrosis develops in the older HIV patients. Besides, HIV stimulates macrophages, platelets and lymphocytes to produce cytokines such as endothelin-1, IL-1β, IL-6 and TNF-α [14]. TNF-α aggravates inflammation and promotes pulmonary fibrosis through NF-κB pathway and by upregulating expression of TGF-β, platelet-derived growth factor (PDGF)-α and PDGF-β [15, 16]. Moreover, NF-κB inhibits fibroblast apoptosis by upregulating Bcl-2 as well as downregulating Bax and caspase 3 to promote pulmonary fibrosis [17]. These proinflammatory cytokines have been shown to play important roles in the development of pulmonary fibrosis, and their upregulated expression may be a vital factor in pulmonary fibrosis induced by HIV (Fig. 2).

### Cytomegalovirus

CMV, a member of the herpesvirus family, persists in a latent state during the primary infection in the host. The complete viral genome resides in the host without production of intact infectious virions, until some stimulus induces reactivation [18]. CMV reactivation of causes serious consequences in non-immunosuppressed critical ill surgical patients and interstitial pneumonia is a frequent manifestation [19, 20]. Yonemaru et al. found increases in CMV immunoglobulin (Ig) G and complement fixation titers with negative CMV Ig M in idiopathic pulmonary fibrosis (IPF) or interstitial pneumonia [21], indicating that CMV infection is associated with the development of pulmonary fibrosis. On HRCT, CMV pneumonia presents bilateral mixed areas of ground-glass attenuation, air-space consolidation, and multiple small nodules [22] (Table 1). CMV reactivation was often associated with fibrinous alveolitis and fibrosis of a mild or moderate extent [23].

In animal study, Cook et al. showed that compared to the control group, the MCMV infected mice had CMV reactivation in 2 weeks after cecal ligation and puncture (CLP), and the mRNA levels of TNF-α, IL-1β, chemokine KC (KC) as well as MIP-2 were altered and pulmonary fibrosis was also developed [18] (Table 2). Li et al. found that MCMV-infected mice did not develop pulmonary fibrosis. Besides, MCMV aggravated pulmonary fibrosis in the bleomycin-treated mice but not in the control mice. They also noticed an increase of vimentin and phospo-Smad2 at protein level [24] (Table 2), indicating that the TGF-β/Smad pathway was activated and Smad2/3 were phosphorylated during this process. Phosphorylated Smad2/3 form a trimer with Smad4, which then translocates to the nucleus and binds to Smad-binding elements to modulate TGF-β target gene expression. TGF-β/Smad pathway increases the expression of pro-fibrotic cytokines such as TIMP-1, Plasminogen Activator Inhibitor-1 (PAI-1), connective tissue growth factor (CTGF), TGF and TGFR1 to activate myofibroblasts. In the meantime, a large amount of collagen is secreted, leading to extracellular matrix (ECM) deposition and development of pulmonary fibrosis [25]. In addition, IL-1β leads to inflammation as well as stimulates the synthesis of collagen and fibrin by fibroblasts to promote pulmonary fibrosis [26] (Fig. 2). Moreover, latent CMV reduces telomere length [27] (Table 2), and short telomeres are considered as a risk factor for pulmonary fibrosis [28].

### Epstein–Barr virus and murine γ-herpesvirus 68

Besides CMV, other herpesviruses are also associated with pulmonary fibrosis, such as EBV and MHV68. Ankermann et al. reported that a 12-month-old immunocompetent girl presented unspecific interstitial lung disease and lung fibrosis after EBV infection. HRCT scan at age 14 months showed segmental air trapping in middle lobe and right atypical lower lobe segment, linear nonseptal (translobular) opacity in middle lobe indicating focal scarring, and peribronchial consolidation in medial lobe segment as well as superior lingula segment. The open-lung biopsy showed uncharacteristic focal interstitial peribronchial infiltration in the left lower lobe, with histiocytes and lymphocytes as well as interstitial fibrosis and increased collagen tissue. After treatment with a combination of inhaled and oral steroids, the child had normal exercise tolerance, and the arterial oxygen saturation was above 93% during resting and post-exercise breathing [29] (Table 1). In the EBV-infected epithelial cells, Malizia et al. observed an increased TGF-β expression and ganciclovir therapy reduced its expression [30]. Sides et al. demonstrated that EBV expressed latent membrane protein (LMP)-1 during the latent phase, which may promote pulmonary fibrosis via NF-κB and ERK pathways [31]. Similarly, Krug et al. found that inhibition of NF-κB signaling diminished the expression of fibrocyte recruiting chemokines MCP-1 and CXCL12 to ameliorate the MHV-68-induced profibrotic events [32]. Mora et al. demonstrated that MHV-68 infection in IFN-γ receptor deficient mice leads to epithelial damage, inflammatory response, collagen accumulation and gradually evolves into progressive interstitial fibrosis. The cytokine profiles and histopathological features of the mice after infection included increased expression of TGF-β and IL-13, imbalance of Th1 and Th2 cytokines, myofibroblast foci, hyperplasia of type II alveolar epithelial cells, altered surfactant proteins and vascular changes. In addition, the elevated expression level of TGF-β coincided with the progression of pulmonary fibrosis [33]. IL-13 induces transdifferentiation of fibroblasts to myofibroblasts via multiple mechanisms: (1) Regulating the JNK signal; (2) Promoting fibroblast proliferation by inhibiting COX expression and prostaglandin E2 (PGE2) production; and (3) Promoting differentiation of fibroblasts via upregulating YY1 (Yin Yang 1) expression in AKT signaling [26]. These results indicate that TGF-β and IL-13 may play important roles in the herpesvirus-induced pulmonary fibrosis. Moreover, the herpesvirus-induced pulmonary fibrosis subsided after treatment with steroids and antiviral therapies [30, 34]. Also, IFN-γ treatment in the early stage of viral infection may be beneficial to reduce the severity of pulmonary fibrosis.

### Influenza virus

Viral pneumonia and immunolocalization of viral antigen in association with diffuse alveolar damage (DAD) are prominent features of infection with 2009 pandemic influenza A (H1N1) virus [35]. H1N1 can rapidly progress to acute respiratory distress syndrome (ARDS) and may induce pulmonary fibrosis. The ARDS secondary to pandemic influenza 2009 manifested DAD and formation of alveolar hyaline membrane, as well as other histologic features included bronchoalveolar pneumonia, interstitial septal thickening, type II pneumonocyte hyperplasia, fibrosis and squamous metaplasia [36] (Table 1). In a HRCT study of 56 H1N1 patients, the most common CT findings were ground glass opacity with or without consolidation during the first week. These abnormalities peaked in the second week and disappeared afterwards, leading to a remarkable reduction in residual disease by 4 weeks or later. And the abnormalities of ground-glass opacities and/or consolidation on initial CT scans tended to resolve completely or resulted in substantial reduced residual disease [37]. It seems that the pulmonary fibrosis induced by H1N1 has the ability to self-rehabilitate, indicating that the underlying mechanism may be different from other lung diseases (Table 1). Mineo et al. demonstrated that pulmonary fibrosis could present different spatial distribution and temporal trend [38]. In a 1-year follow-up study, Saha et al. demonstrated that patients who recovered from H1N1-induced ARDS still had the incidence of pulmonary fibrosis. They also found that the patients could recover from pulmonary fibrosis with the combination therapy of pirfenidone, azithromycin and prednisolone [39]. Pulmonary fibrosis in the H1N1 patients may be related to the increased activity of fibroblasts in the post-inflammatory repair pathways. During this process, TGF-β and collagen play important roles. A number of studies showed an appreciable rise of TGF-β in the patients with pulmonary fibrosis. Wen et al. found that all the cytokines were nearly unchanged during the observation period, except TGF-β. Also, they showed that TGF-β was overproduced in the severe patients but not in the mild patients [40], indicating that the pulmonary fibrosis may be related to the disease severity. In addition, the animal models of H1N1 infection showed similar outcomes. Shatskaya et al. found that H1N1 infection enhanced the expression of TGF-β and Smad-2 by macrophages and alveolar cells, indicating that H1N1 induced pulmonary fibrosis possibly by activating TGF-β/Smad pathway [41] (Table 2, Fig. 2). Moreover, Drakopanagiotakis et al. demonstrated that increased apoptosis of alveolar epithelial cells and decreased apoptosis of fibroblasts play important roles in the pathogenesis of pulmonary fibrosis [42]. Roberson et al. found that influenza A virus infection induces endoplasmic reticulum (ER) stress and elicits multiple responses such as caspase-12 dependent epithelial apoptosis and JNK1-dependent TGF-β expression [43]. These results indicated that influenza infection may activate the TGF-β/Smad pathway and ER stress to promote pulmonary fibrosis.

### Avian influenza virus

Avian influenza virus has also been reported to induce pulmonary fibrosis. Here we mainly discuss the pulmonary fibrosis induced by H7N9 and H5N1. All survivors of H5N1 and H7N9 were found to have lung involvement by the HRCT images, with ground-glass opacities and lobar consolidation as the most typical chest CT findings, which is possibly due to the DAD with proteinaceous exudates, occasional cytomegaly and intra-alveolar hemorrhage [44–46] (Table 1). In the early stage of H5N1 infection, the lesion expands rapidly within a few days, leading to disseminated exudation changes in both lungs. During climax phase, most of the lung areas are involved. For severe H5N1 patients, recovery is a long process, pulmonary lesions persist even after clinical symptoms have disappeared [44]. In the same study, the 12th and 24th month follow-up CT of severe H5N1 patients still demonstrated ground-grass shadows, apparent reticular pattern, irregular linear opacities, interlobular septal thickening and intra-lobular lines. The 12th month follow-up CT of another patient showed only minimal residual fibrous lines, indicating that the H5N1-induced pulmonary fibrosis was reversible. The recovery process of patients with severe H7N9 infection also lasts long. A follow-up examination at 32 weeks after onset CT images showed different extents of secondary fibrosis and traction bronchiectasis. Additionally, it is worth noting that the time to recovery from lymphopenia was highly consistent with the time to return to normal in the chest CT findings, indicating that lymphopenia is a feature of H7N9 and recovery from it contributes to clearance of the virus and improvement of condition [45]. Autopsy of the patients with H5N1 showed the presence of organizing DAD with interstitial fibrosis [47]. Similar manifestation can also be seen in autopsy reports of H7N9-infected patients, including hyperplasia and shedding of type II pneumocytes, severe pulmonary fibrosis, numerous lymphocytic infiltrates and only few neutrophilic infiltrates, consistent with the radiological findings [48]. Similar human pathophysiology was observed in the H5N1-infected mice. Qiao et al. demonstrated that the H5N1-infected mice developed diffuse pneumonia with inflammatory cell infiltration 7 days post infection. On the 30th day, different degrees of pulmonary fibrosis were observed in most mice [49]. Studies demonstrated that H5N1 infection significantly increased the secretion of pro-fibrotic factors such as TNF-α, IL-6, fibroblast growth factor (FGF) and epidermal growth factor (EGF) in mice [50, 51] (Table 2). Additionally, angiotensin-converting enzyme 2 (ACE2) expression was decreased in H5N1 and H7N9 infected patients and it induced an elevated angiotensin II (Ang II) expression level [52, 53]. Ang II and ACE2 are components of Renin-angiotensin system (RAS), which regulates the occurrence of pulmonary fibrosis. Ang II binds to Ang II type 1 receptor (AT1R) and increases the expression of TGF-β, NF-κB and PAI-1 to promote pulmonary fibrosis. Besides, Ang II significantly increases NOX4 level and reactive oxygen species (ROS) production in lung fibroblasts, stimulating fibroblast migration and α-collagen I synthesis through the RhoA/Rock pathway. Contrarily, ACE2 produces Ang1-7 which reduces inflammation by decreasing secretion of NF-κB and inhibiting the NOX4-derived ROS-mediated RhoA/Rock pathway to protect the lung from injury and inhibit fibrosis. ACE2 also has an inhibitory effect on Ang II [17, 54]. Ang II induces apoptosis of alveolar epithelial cells through mitochondrial pathway and inhibits apoptosis of fibroblasts through NF-κB pathway [55] (Fig. 2). These results indicated that avian influenza infection decreased the expression of ACE2 as well as its inhibitory effect on Ang II, possibly shifting the balance of RAS toward fibrosis promoting direction. Zou et al. also demonstrated that human recombination ACE2 effectively protected the lung of H5N1-infected mice by reducing viral replication [52]. Kuba et al. found that ACE inhibitor attenuates epithelial apoptosis, interstitial fibrosis, and collagen deposition in the bleomycin-induced pulmonary fibrosis mice [56].

### MERS-CoV

MERS-CoV belongs to the Coronavirus genus, and its receptor is dipeptidyl peptidase 4 (DPP4). The symptom appeared in 2–14 days after MERS-CoV infection, mild patients had low-fever, runny nose, sore throat and muscle aches, while the severe patients developed acute respiratory distress syndrome. From the chest radiographs and CT scans of severe patients, multi-lobar airspace disease, ground-glass opacities and pleural effusions were observed [57] (Table 1). In the autopsy of a patient infected with MERS-CoV, the predominant pulmonary histologic pattern was DAD with denuding of bronchiolar epithelium, prominent hyaline membranes, alveolar fibrin deposits and type II alveolar cells hyperplasia [58]. Das et al. showed that a substantial portion of patients who recovered form MERS-CoV infection developed pulmonary fibrosis, and the development was fairly rapid with the mean ± SD, median, and range of pulmonary fibrosis progression after discharge being 82.4 ± 66 days, 44 days, and 32–230 days, respectively. In addition, the morbidity and extent of pulmonary fibrosis were related to the severity and infection period of MERS-CoV infection [59]. MERS-CoV has a much broader tissue tropism than SARS-CoV and resembles SARS-CoV in suppressing interferon production [60, 61]. As compared to the SARS-CoV infection, MERS-CoV infection induced significantly higher expression levels of IL-8, IL-12, IP-10/CXCL-10 and MCP-1/CCL-2 [60]. Yeung et al. demonstrated that MERS-CoV-induced apoptosis by increasing the expression of Smad7 and FGF2 [62]. Kim et al. showed that histopathological features of pulmonary fibrosis were observed in MERS-CoV-infected hDPP4-transgenic (hDPP4-tg) mice, including alveolar septal thickening, inflammatory monocyte infiltration, macrophage polarization, acute pulmonary inflammatory response, as well as increased expression of pro-inflammatory factors and pro-fibrotic factors, such as TNF-α, IL-1β, TGF-β, type I collagen and type III collagen [63] (Table 2). Falzarano et al. demonstrated that MERS-CoV infection up-regulated the receptors for IL-2, IL-4, IL-6, IL-17, IL-22, IL-27 and down-regulated the receptors for IL-1, IL-12, IL-18 and IL-20 in marmoset [64]. They also noticed the down-regulation of IFN-γ and its receptor at multiple time points, but no expression of IFN-β. The absence of interferon and secretion of pro-inflammatory cytokines may aggravate lung injury and promote pulmonary fibrosis. IL-4, IL-6 and IL-17 also contribute to pulmonary fibrosis by promoting collagen synthesis and transdifferentiation of fibroblasts to myofibroblasts [26].

### SARS-CoV

SARS is an acute infectious disease with high morbidity and mortality caused by coronavirus SARS-CoV [65]. SARS-CoV also belongs to the Coronavirus genus. The symptom of the SARS-CoV infection includes persistent high fever, chills, malaise, myalgia, headache and dry cough. Most patients recovered in 1–2 weeks, however, around one-third patients developed ARDS after SARS-CoV infection and had a high mortality [66]. In the acute phase of the SARS development (7–10 days post infection), the lung showed the features of DAD including extensive edema, hyaline membrane formation, alveolar collapse, and alveolar epithelial desquamation. In the next phase of SARS development (10–14 days post infection), the feature of fibrosis appeared. As the development of the SARS-CoV infection lasted more than 2–3 weeks, the extent of pulmonary fibrosis also got further increased [67, 68] (Table 1). The fibrotic lesion can also be found in the autopsy of fatal cases, and the degree of pulmonary fibrosis is related to the duration of SARS-CoV infection. The virus persisted in the lung tissue of the autopsy, indicating continued damage to the lung after infection [68]. Pulmonary fibrosis occurs not only during the period of the SARS-CoV infection, but also during the recovery period. One month after discharge, 40 out of 51 patients with positive SARS-CoV IgG results and abnormal lung diffusion were found pulmonary fibrosis by HRCT [69]. Moreover, the patients recovered from the SARS-CoV infection, especially those with old age and severe disease course, have a higher morbidity of pulmonary fibrosis and worse lung function as compared to the uninfected people [70]. A 15-year follow-up study of SARS patients showed that 27 out of 71 patients exhibited ground-glass opacities or cord-like consolidation, indicating that the lung injury lasted for a long time [71] (Table 1). In addition, pulmonary fibrosis was found spontaneously resolved in some patients, indicating that the pulmonary fibrosis induced by SARS-CoV infection is reversible, but the mechanism is unknown [69, 72] (Table 1). Although the exact mechanism of SARS-CoV-induced pulmonary fibrosis remains unspecified, various animal studies suggested that TGF-β and ACE2 may play important regulatory roles. As mentioned above, TGF-β promotes pulmonary fibrosis through multiple mechanisms. ACE2 has been proved to be anti-fibrotic and exerts a protective effect in the lung by negatively regulating Ang II (Fig. 2). Rockx et al. found that ACE2 expression was significantly decreased in the SARS-CoV infected mice and associated with mortality. Although pulmonary fibrosis was not examined in this study, the expression of pro-fibrotic cytokines was significantly increased [73] (Table 2). Zhao et al. demonstrated that N protein not only increases the transcriptional responses of TGF-β by promoting Smad-p300 complex formation, but also enhances the expression of PAI-1 and attenuate Smad3/Smad4-mediated apoptosis [74]. Similarly, Gralinski et al. found that SARS-CoV infection significantly elevated transcripts of pro-inflammatory cytokines such as IL-1β, TNF-α, IL-6 and pro-fibrotic TGF-β, CTGF and PDGF in mice [75] (Table 2). They also noticed an increased expression of PAI-1, which induces alveolar type II cell senescence, collagen deposition and secretion of pro-fibrotic mediators to promote pulmonary fibrosis [75–77] (Fig. 2). Page et al. found that SARS-CoV infection caused Th2 bias and differentiation of alternatively activated macrophages (AAM) in the lung of the STAT1 knockout mice, leading to overactivity of the macrophages in the damaged site and possibly promoted pulmonary fibrosis [78]. In addition, Th2 bias aggravates pulmonary fibrosis through pro-fibrotic and pro-inflammatory cytokines [79].

### SARS-CoV-2

With the global outbreak of COVID-19, pulmonary fibrosis as one of the complications of SARS-CoV-2 infected patients, deserves more attention [80]. It is associated with disease severity, age, ARDS, longer in-hospital stays, tachycardia, non-invasive mechanical ventilation and higher initial chest CT score [2, 82, 83]. In fatal cases of SARS-CoV-2 infection, pulmonary fibrosis was generally present at autopsy [84]. An elderly female patient with no history of lung disease died of severe bilateral pulmonary fibrosis after elimination of COVID-19 infection. CT showed extensive pulmonary fibrosis on both sides, while microscopic examination showed fibrosis with honeycomb-like remodeling and bronchial metaplasia [85]. A 6-month follow-up study showed that pulmonary fibrosis developed not just in the course of SARS-CoV-2 infection, but also in the post-discharge stage of more than one-third of the infected patients who survived severe COVID-19 pneumonia [2]. In another follow-up study, thin-section chest CT showed that pulmonary fibrosis developed in COVID-19 patients could reverse in about a third of 397 patients 120 days after the onset [86] (Table 1). Although pulmonary fibrosis would be a major complication in patients healing from SARS-CoV-2 infection, current data is unclear on the prevalence of this sequela and longer follow-up studies with larger sample size are needed. The most common patterns on the chest CT of COVID-19 patients were ground-glass opacity and bilateral patchy shadowing [81]. Increase in the ground glass density patches and fibrous stripes was observed in a span of 3–14 days on a follow-up CT [87]. Histopathological examination of the lung biopsy tissues revealed bilateral acute changes with DAD, reactive type II pneumocyte and macrophage hyperplasia, patchy inflammatory cellular infiltration and loose interstitial fibrosis [88] (Table 1). All the radiological and histopathological evidence indicated that the SARS-CoV-2 infection may be a trigger of pulmonary fibrosis, and excessive immune response caused by cytokine storm is possibly involved. Besides, Hu et al. found that the baseline levels of IFN-γ were negatively associated with the increase of fibrosis volume in COVID-19 at discharge [89]. Valdebenito et al. noticed an increased infiltration of macrophages in the lungs of the autopies, as well as massive loss of alveolar cells, and proliferation of fibroblasts [90].

Persistent lung injury is considered as a main cause of pulmonary fibrosis. During SARS-CoV-2 infection, the virus replicates rapidly and binds to the ACE2 receptor on the surface of lung epithelial cells. Then the alveolar type II cells are infected, leading to alveolar injury and impaired gas exchange. Macrophages are activated and accumulated after injury to reduce inflammation. However, the adverse conditions such as the vigorous response of pro-inflammatory cytokines and chemokines, the continued proliferation of viruses, as well as the destruction of a large number of lung cells lead to the polarization of M1 macrophages and further promote inflammation. As the injury progresses, a series of host inflammatory responses, such as excessive production of cytokines and a large influx of inflammatory cells, can be observed at the site of injury [93–95]. Harrison et al. showed that SARS-CoV-2 infection increased the expression of IFN-I, IFN-II, TNF-α, IL-1β, IL-6, IL-8, IL-18, GM-CSF, CXCL9, CXCL10, CXCL11, CCL-2 and MIP1a in BALF and serum, leading to sustained lung injury [93]. Eventually, severe scarring and fibrosis of lung tissue are caused by collagen deposition. Chen et al. found that compared to moderate patients, severe patients expressed higher level of IL-2R, IL-6, IL-10, and TNF-α [96]. IL-6 has been shown to promote pulmonary fibrosis through the STAT pathway [97] (Fig. 2), so IL-6 may be one of the important factors for SARS-CoV-2-induced pulmonary fibrosis. Xu et al. found that the mRNA transcripts of pro-fibrotic cytokines such as TGF-β, fibronectin 1 (FN1) and CTGF were significantly increased in the alveolar epithelial cells of the patients with pulmonary fibrosis induced by SARS-CoV-2 [98]. SARS-CoV-2 infection also leads to abnormal expression of pulmonary fibrosis related proteins, for example, MMP2, MMP8 and cathepsin were found to be upregulated and E-cadherin was downregulated [99]. Moreover, just like SARS-CoV, SARS-CoV-2 infection downregulates ACE2 expression, disrupts the balance of RAS, and may promote the progression of RAS towards inflammation and fibrosis [100].

In animal models, SARS-CoV-2 infection also induced expression of pro-fibrotic cytokines and occurrence of pulmonary fibrosis [91, 92] (Table 2). Although the specific mechanism needs further study, SARS-CoV-2-induced pulmonary fibrosis has many similarities with pulmonary fibrosis induced by other viruses. According to the existing studies, the development of pulmonary fibrosis in the patients with SARS-CoV-2 infection is accompanied by the increased expression of cytokines such as TGF-β, TNF-α, IL-1β, IL-6 and IL-13. Besides, some studies have reported that SARS-CoV-2 infection not only decreases ACE2 expression but also inhibits autophagy. All these indicate that TGF-β, TNF-α, IL-1β, IL-6, IL-13, ACE2 and autophagy inhibition may be the key factors in pulmonary fibrosis induced by SARS-CoV-2.

## Conclusions and future perspective

Though the mechanisms underlying the virus infection induced pulmonary fibrosis have not been fully elucidated, commonalities can be found. Abnormal expression of pro-fibrotic TGF-β can be seen in most virus-induced pulmonary fibrosis. In the meantime, abnormal immune response induced by the virus also plays a vital role in the formation of pulmonary fibrosis. The lung is damaged by viral infection and often shows inflammatory infiltrates, while macrophages gather and polarize at the damaged site, releasing a large number of pro-inflammatory and pro-fibrotic cytokines/factors, such as IL-1β, IL-6, IL-17 and TNF-α. Meanwhile, the type 2 immune response is activated and results in an increased secretion of Th2 cytokines such as IL-4, IL-5, and IL-13. These cytokines/factors not only cause sustained damage to the lung, but also induce formation of pulmonary fibrosis. Besides, virus infection often causes a decreased expression of ACE2, shifting the balance of RAS towards pro-fibrotic direction.

It is also worth noting that virus infection is often accompanied by autophagy inhibition, which leads to massive proliferation of fibroblasts and promotes formation of pulmonary fibrosis. Viral membrane-associated papain-like protease 2 (PLP2-TM) produced by both SARS-CoV and MERS-CoV induces autophagosome formation, and blocks the fusion of autophagosomes with lysosomes [101]. SARS-CoV-2 has also been reported to prevent autophagy progression by hindering autophagosome-lysosome fusion [102]. HIV protein Nef inhibits the autophagy process by interacting with the autophagy regulator Beclin-1, thus protecting HIV from degradation [103]. Besides, CMV encodes TRS1 and IRS1 proteins to inhibit autophagy process [104, 105]. MHV-68 M11 encodes proteins to antagonize autophagy [106]. Previous studies also showed that abnormal autophagy activity leads to the occurrence of pulmonary fibrosis. Hill et al. suggested that autophagy inhibition induces EMT of alveolar epithelial cells and contributes to pulmonary fibrosis via aberrant epithelial-fibroblast crosstalk [107]. Moreover, virus infection increases ROS expression and secretion of mTOR, which inhibits autophagy and promotes lung fibroblast proliferation via PI3K-Akt-mTOR pathway. ER stress does not only augment proliferation and migration of fibroblasts but also impairs protein homeostasis in alveolar epithelial cells, promoting pulmonary fibrosis [108]. All these suggested that decreased autophagy may be one mechanism for pulmonary fibrosis induced by viral infection. In this sense, autophagy modulators may be effective to treat pulmonary fibrosis induced by viruses including SARS-CoV-2.

It’s worth noting that the pulmonary fibrosis post virus infection may not be a direct result of infection, and other factors such as ventilator-induced injury may come into play as well. Many severe patients with viral infection use mechanical ventilation during treatment and the follow-up study showed that patients with longer period of mechanical ventilation had a higher probability of developing pulmonary fibrosis and a worse prognosis [2, 19]. Diem et al. showed that alveolar cells under mechanical stress can produce signal molecules to communicate with neighboring cells and promote fibrotic response [109].

Similar to SARS-CoV and other viruses, SARS-CoV-2 also causes long-term lung damage and persistent pulmonary fibrosis. In order to make patients better prognosis, timely anti-viral and anti-fibrosis therapy is necessary. The safety, dosage and intervention time of traditional anti-fibrosis drugs (i.e., corticosteroids) need to be tested. Besides, IL-6 neutralizing antibody and human recombinant ACE2 have already been proved to inhibit bleomycin-induced pulmonary fibrosis in mice. Whether these interventions are effective in treating pulmonary fibrosis induced by SARS-CoV-2 awaits further study.

The mechanisms underlying the virus-induced pulmonary fibrosis needs to be studied in depth. Comparing pulmonary fibrosis induced by virus with pulmonary fibrosis of other types may reveal its distinct features and mechanisms. Moreover, systematic comparison of the lung genome and cytokine profile between the virus-infected animals and the control animals at different stages post infection may provide not only biomarkers and therapeutic targets, but also strategies for early diagnosis and monitoring the fibrosis progression.

There are still many remaining questions awaiting for further study. For instance, pulmonary fibrosis induced by some virus resolves spontaneously while some other may persist and require drug treatment. Long-term follow-up study is necessary. At present, there is  a lack of follow-up study for pulmonary fibrosis induced by some viruses. Periodic HRCT examination of patients would be helpful to determine whether pulmonary fibrosis is reversible or not and how long it lasts. Viruses usually damage the lung rapidly in the early stage of infection. Therefore, anti-viral therapy combined with anti-fibrotic therapy may be better to be carried out at early stage. To this end, clearer standards are needed to determine the type, developmental stage and features of the virus-induced pulmonary fibrosis. Besides, fibrosis is a dynamic and complex process, coupled with the heterogeneity of patients, even if triggered by the same virus, it could vary greatly among patients. In order to carry out a more comprehensive assessment of the virus-induced pulmonary fibrosis, combination of multiple means, such as HRCT, pathological methods and molecular diagnosis are needed.

In sum, defining the clinical symptoms, histopathological and molecular features, as well as exploring the underlying mechanisms are of great significance for the early diagnosis and treatment of the virus-induced pulmonary fibrosis.

## Data Availability

Not applicable.

## Abbreviations

ACE2: Angiotensin-converting enzyme 2
Ang II: Angiotensin II
ARDS: Acute respiratory distress syndrome
ATL: Adult T-cell leukemia lymphoma
BALF: Bronchoalveolar lavage fluid
CMV: Cytomegalovirus
CTGF: Connective tissue growth factor
DAD: Diffuse alveolar damage
DPP4: Dipeptidyl peptidase 4
EBV: Epstein–Barr virus
ECM: Extracellular matrix
EGF: Epidermal growth factor
EMT: Epithelial-mesenchymal transition
FGF: Fibroblast growth factor
HIV: Human immunodeficiency virus
HTLV: Human T-cell leukemia virus
MCP-1: Monocyte chemoattractant protein 1
MERS: Middle east respiratory syndrome
MHV68: Murine γ-herpesvirus 68
MMP: Matrix metalloproteinases
PAI-1: Plasminogen activator inhibitor-1
PDGF: Platelet-derived growth factor
RAS: Renin-angiotensin system
SARS: Severe acute respiratory syndrome
TGF-β: Transforming growth factor-β
TIMP: Tissue inhibitor of metalloproteinases
TNF-α: Tumor necrosis factor-α
